# Electrochemical Assay of Human Islet Amyloid Polypeptide and Its Aggregation

**DOI:** 10.3390/s8095987

**Published:** 2008-09-25

**Authors:** Nandi Zhou, Zhenyu Chen, Dongmei Zhang, Genxi Li

**Affiliations:** 1 Laboratory of Biosensing Technology, School of Life Science, Shanghai University, Shanghai 200444, P.R. China; 2 Department of Biochemistry and National Key Laboratory of Pharmaceutical Biotechnology,Nanjing University, Nanjing 210093, P.R. China

**Keywords:** Human islet amyloid polypeptide, bioelectrochemistry, aggregation, square wave voltammetry

## Abstract

Square wave voltammetry is used in this work to detect human islet amyloid polypeptide (hIAPP) by using the oxidized signal of the tyrosine residue in hIAPP. A detection limit of 1×10^-6^ M for hIAPP has been obtained. A kinetic study of the aggregation process has been carried out according to the relationship between the anodic peak current in the square wave voltammograms of hIAPP and the incubation period. The results show that the nucleation starts in the first hour of incubation and then, during the next two hours, aggregation may occur rapidly. hIAPP can therefore be monitored with a label-free electrochemical method with low detection limit and high sensitivity. This electrochemical method can be also utilized to study the kinetics of hIAPP aggregation, and it may be also employed to study the conformational changes of the polypeptide.

## 1. Introduction

Some special proteins or polypeptides aggregating into cytotoxic oligomers, polymers or fibrils *in vivo* have been discovered to be related to cell degeneration and the pathogenesis of many uncurable diseases such as Alzheimer's disease, type-2 diabetes, Parkinson's disease, etc., which are called protein aggregation diseases. Meanwhile, because of the aggregation, normally soluble proteins will be converted into insoluble amyloid, which consists of fibrils rich in β-sheet structures and has characteristic dye-binding properties [1-4].

Among these kinds of proteins, human islet amyloid polypeptide (hIAPP) or amylin is a 37-residue peptide containing an amidated C-terminal and a disulfide linkage between cysteine residues 2 and 7, synthesized in the pancreas and co-secreted with insulin [5-7]. It has been suggested that hIAPP may play an important role in glucose homeostasis together with insulin [8-9]. Though hIAPP is itself a soluble polypeptide, its flexible conformation has a tendency to misfold into insoluble, cytotoxic fibrils through a complicated multistep nucleation-aggregation process [10-12]. Moreover, the oligomers and polymers of hIAPP are proven to be cytotoxic and linked to the progressive deterioration of pancreatic β-cell and pathogenesis of type-2 diabetes [13-16]. Therefore, an assay of hIAPP and its aggregation is highly required [17-25].

The previous assay methods are basically based on the techniques of microscopy and spectroscopy, such as atomic force microscopy, circular dichroism spectroscopy, fluorescence assay, etc. Electrochemical techniques are known to be useful to study the oxidation reactions of tyrosine, tryptophan and related peptides. Electrochemical methods have also been adopted to monitor the folding and unfolding of proteins and aggregation of peptides [26-27]. An intrinsic redox-active amino acids-based detection method has been used to fabricate label-free electrochemical protein sensors [28]. Therefore, based on our electrochemical studies on proteins and the related biological processes [29-32], we have studied the aggregation process of hIAPP with electrochemical method, and have proposed an electrochemical assay method for this protein aggregation. A kinetic analysis has also been performed.

## 2. Results and Discussion

**Figure 1.**
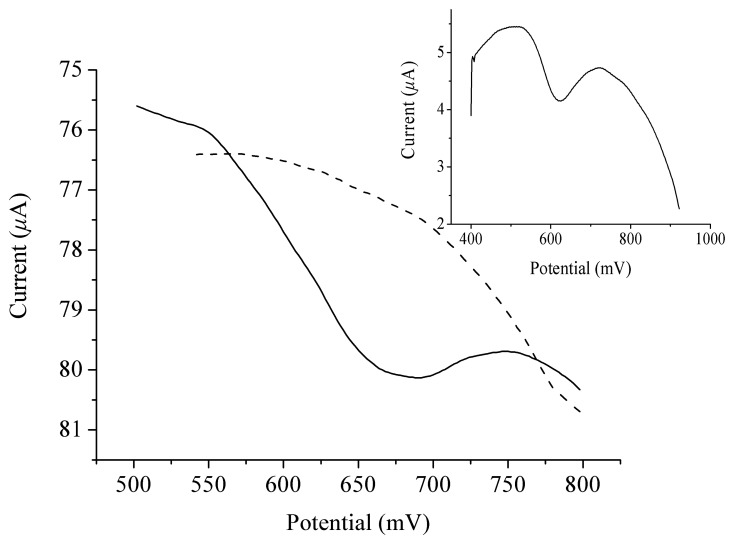
Square wave voltammograms of hIAPP at the concentration of 2.5×10^-5^ M (solid line) and 0 M (dashed line). Inset is the square wave voltammogram of tyrosine solution at the concentration of 2×10^-6^ M.

It has been known that tyrosine can be oxidized irreversibly giving an anodic peak in the corresponding voltammogram. As shown in the inset of Figure 1, when SWV is performed, an oxidation wave with a peak potential of 650 mV appears in the voltammograms of tyrosine solutions. We here use SWV rather than cyclic voltammetry because SWV gives better sensitivity. When cyclic voltammetry is used, a 1×10^-5^ M tyrosine solution must be used in order to obtain a nice observable peak, whereas in the SWV experiements, a 2×10^-6^ M tyrosine solution worked well.

Tyrosine residues are widespread in proteins or polypeptides. In hIAPP, although it is a 37-residue peptide containing only one tyrosine residue, this residue is at its C-terminal and therefore it may endow the peptide with oxidable properties on an electrode surface. In fact, as is shown in Figure 1, an oxidation peak at 650 mV derived from the oxidation of the tyrosine residue in hIAPP can indeed be observed and therefore, a possible method to assay hIAPP with an electrochemical technique may be developed.

**Figure 2.**
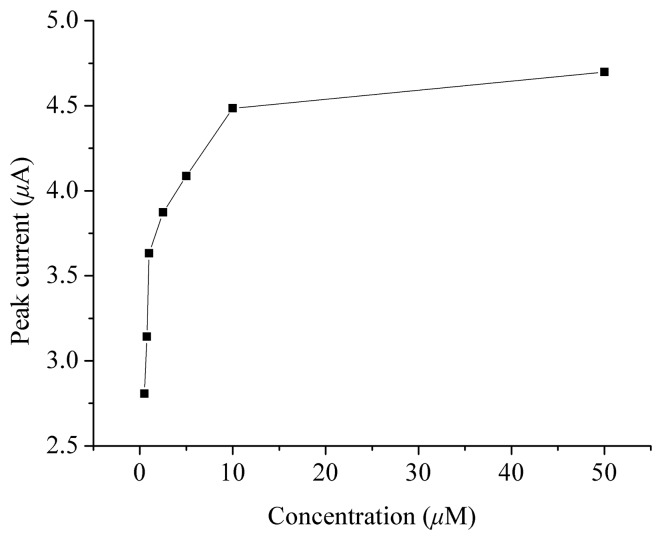
Relationship between the oxidation peak current of hIAPP and its concentration.

The relationship between the oxidation peak current in the voltammogram and the concentration of hIAPP has been examined. The results show that the peak current increased as the concentration of hIAPP increased, although the relationship is not linear (Figure 2). Therefore, it might be possible to develop an electrochemical method to study the aggregation process of this polypeptide, because with aggregation, less and less tyrosine residues can be oxidized to give an electrochemical signal, so the oxidation peak will be smaller and smaller.

**Figure 3.**
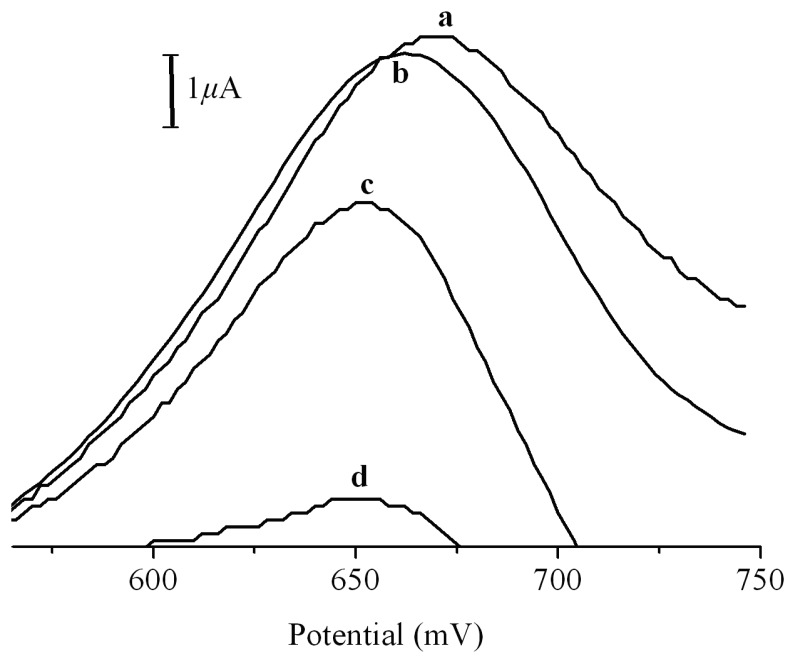
Square wave voltammograms of 5.1×10^-5^ M hIAPP incubated at 37 °C for (a) 0 h, (b) 1 h, (c) 2 h and (d) 5 h.

It should be mentioned that hIAPP may dissolve from the electrode surface into the test solution during the scanning of the modified electrode. Therefore, the modified electrode should not be kept in solution when not in use, and the electrode cannot be used for a long time. Nevertheless, since the purpose of this study was to develop an assay method, and not for quantitative analysis or for a sensor fabrication, the short duration of the usage of the modified electrode can still meet the requirements of this work and in practice, the stability of the electrode and reproducibility of the measurements were both satisfactory. We have performed all the experiments to obtain the data in Figure 2 several times, and the experimental results reveal that the relative standard deviation (R.S.D.) is between 0.86% and 3.80%. On the other hand, since the duration of the drying of hIAPP on the electrode surface is about 30 min, and the oxidation peak current will be nearly unchanged in the first hour during the aggregation of hIAPP (Figure 3), it can be believed that the aggregation of hIAPP will not affect the detection of hIAPP.

The tyrosine oxidation-based electrochemical method can be further utilized to study the kinetic process of aggregation of hIAPP. Figure 3 shows the square wave voltammograms of 5.1×10^-5^ M hIAPP incubated at 37 °C for different times. Obviously, the oxidation peak declines as the incubation period increases. This is reasonable, since the C-terminal tyrosine residue is accessible to the electrode surface and easily oxidized if it is in the soluble form. When hIAPP converts to its insoluble β-sheet fibrillar aggregation state, the tyrosine residues become somewhat inaccessible to electrode surface, which thus causes the observed decline of the oxidation peak.

**Figure 4.**
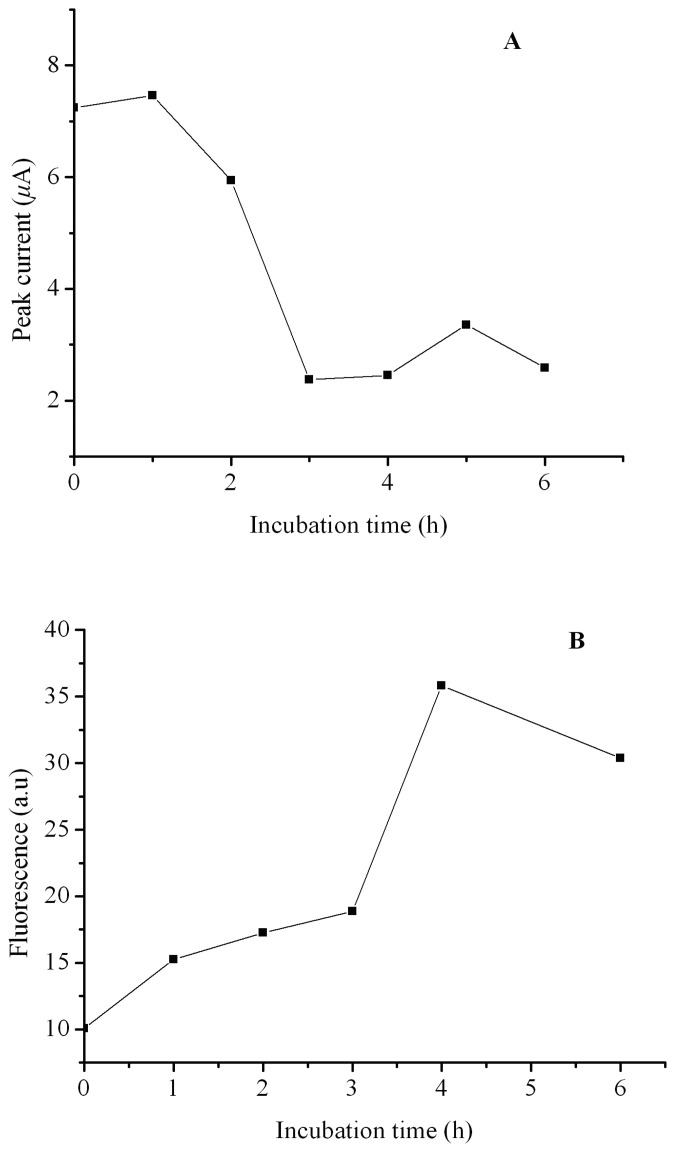
(A) Relationship between the oxidation peak current in the square wave voltammograms of hIAPP and the incubating period. (B) Relationship between the fluorescent intensity of thioflavin-T treated hIAPP solution and the incubating period. Others same as in Figure 3.

Figure 4(A) shows the relationship between the oxidation peak current and the incubation time (R.S.D.: 0.73% ∼ 2.02% for three measurements). During the incubation period the peak current is nearly unchanged from 0 to 1 h. This result may be due to a multistep nucleation-aggregation and concentration-dependent process that proceeds via a conformational transition of mainly random coil hIAPP into β-sheet-containing amyloid aggregates. At the beginning of the aggregation process, a nucleation period during which soluble oligo- and multimeric hIAPP are formed is necessary [10, 16, 33, 34]. This period may have little influence on the oxidability of the tyrosine residue in C-terminal of hIAPP. However, as the nucleating period is finished and multimeric hIAPP seeds are formed, a conformational transition process starts, accompanied by a rapid decrease of the free tyrosine residues. As a result, as shown in Figure 4(A), the peak current declined rapidly during the incubation period from 1 to 3 h. After the three hour incubation period, the peak current of hIAPP reaches a minimum and can be hardly changed with a further prolonged incubation period, which suggests that hIAPP has completed changed its conformation from a soluble monomer to amyloid aggregates within the three hours.

We have further compared the electrochemical analysis with a referenced thioflavin-T based fluorescent assay. Thioflavin-T and its derivatives can bind to amyloid fibrils in a specific, regular fashion, thus the increase in thioflavin-T fluorescence emission has been broadly used as a specific and quantitative assay for fibril formation. Figure 4(B) shows the relationship between fluorescent intensity of thioflavin-T treated hIAPP solution and the incubation period. Comparing Figure 4(B) with Figure 4(A), we can see that these two assay methods may present a similar result, but suggesting a different mechanism. Thioflavin-T based fluorescent assay reflects exactly the content of amyloid fibril, however, the electrochemical assay may be more informative in studying the degree of conformational change of hIAPP during fibril formation.

## 3. Experimental Section

### 3.1 Chemicals

Human islet amyloid polypeptide, with the amino acid sequence Lys-Cys-Asn-Thr-Ala-Thr-Cys-Ala-Thr-Gln-Arg-Leu-Ala-Asn-Phe-Leu-Val-His-Ser-Ser-Asn-Asn-Phe-Gly-Ala-Ile-Leu-Ser-Ser-Thr-Asn-Val-Gly-Ser-Asn-Thr-Tyr was obtained from Sigma Chemical Company. L-tyrosine and thioflavin-T were also purchased from Sigma. Other reagents were of analytical grade. Water was purified with a Milli-Q purification system to a specific resistance (>18 MΩ/cm) and used to prepare all the solutions.

### 3.2 Apparatus

Electrochemical experiments were carried out with a model 263A Potentiostat/Galvanostat (EG&G, USA) and a three-electrode system. The working electrode was a modified pyrolytic graphite electrode (PGE). A saturated calomel electrode (SCE) was used as the reference electrode and all potentials reported in this work were versus SCE. A platinum wire electrode served as the counter electrode. Fluorescence measurements were performed on a model RF5301PC spectrofluorometer (Shimadzu, Japan) using 5 nm slit widths for both excitation and emission measurements.

### 3.3 Preparation of hIAPP solution

0.1 mg hIAPP was first dissolved in dimethyl sulphoxide (DMSO, 20 μL). The solution was then added to a 0.05 M phosphate buffer (pH 7.0, 480 μL), thus a stock solution of hIAPP with the concentration of 0.2 mg/mL (5.1×10^-5^ M) was prepared. To obtain hIAPP solutions of different concentrations, the stock solution was diluted with the corresponding volume of phosphate buffer.

### 3.4 Electrochemical measurement

The substrate PGE was first abraded with successively finer grades sand papers. Then, it was polished to a mirror smoothness with alumina powder (Al_2_O_3_) of various particle sizes (0.3 and 0.05 μm) on silk. Finally, the electrode was thoroughly washed by ultrasonicating in both ethanol and water for 5 min. hIAPP with different concentrations (10 μL) was evenly spread on the surface of the above pretreated PGE. After drying for about 30 min, the modified electrode was then ready for electrochemical detection. To study the kinetic process of aggregation, 5.1×10^-5^ M hIAPP solution was first incubated at 37 °C for some period. Then, hIAPP (10 μL) with different incubation times, i.e., with different degrees of aggregation, was evenly spread on the surface of the PGE. After drying, the modified electrode could be then used as the working electrode.

Square wave voltammetry (SWV) was adopted to detect hIAPP and to study the aggregation process. SWV experimental conditions were 2 mV step height, 50 mV pulse height, and 100 Hz frequency. Supporting buffer was 0.05 M phosphate solution (pH7.0). Voltammograms were recorded as soon as PGE was inserted into the supporting buffer solution in order to minimize the dissolution of hIAPP from the surface of the working electrode.

### 3.5 Fluorescence study

Fluorescence spectrometry was also used to study the kinetic process of hIAPP aggregation for comparison. Accordingly, 5.1×10^-5^ M hIAPP solution was also incubated at 37 °C for some periods. Then, hIAPP (15 μL) with different aggregation times was sampled and added into 2.5×10^-5^ M thioflavin-T solution (3 mL). After shaking, fluorescence spectrum of the mixed solution was recorded at the excitation wavelength of 445 nm, and the intensity at emission wavelength of 485 nm was used to analyze the degree of aggregation.

## 4. Conclusions

In conclusion, hIAPP can be monitored with an electrochemical method by the oxidation peak of tyrosine residue at the C-terminal. Compared with other techniques, this method is fast and label-free, and it can detect concentrations of hIAPP as low as 1×10^-6^ M. This electrochemical method can be also utilized to study the kinetics of hIAPP aggregation. Compared with referenced thioflavin-T fluorescent assay, this method may be also employed to study the conformational changes of the polypeptide, besides the aggregation process. Based on this study, investigation of type-2 diabetes and the drug screening might be carried out with electrochemical method in the future.

## References

[1] Selkoe D.J. (2003). Folding proteins in fatal ways. Nature.

[2] Glenner G.G. (1980). Amyloid deposits and amyloidosis: the β-fibrilloses (first of two parts). N. Engl. J. Med..

[3] Glenner G.G. (1980). Amyloid deposits and amyloidosis: the β-fibrilloses (second of two parts). N. Engl. J. Med..

[4] Dobson C.M. (2003). Protein folding and misfolding. Nature.

[5] Cooper G., Willis A.C., Clark A., Turner R.C., Sim R.B., Reid K.B. (1987). Purification and characterization of a peptide from amyloid-rich pancreases of type 2 diabetic patients. Proc. Natl. Acad. Sci. USA.

[6] Westermark P., Wernstedt C., Wilander E., Sletten K. (1986). A novel peptide in the calcitonin gene related peptide family as an amyloid fibril protein in the endocrine pancreas. Biochem. Biophys. Res. Commun..

[7] Westermark P., Wernstedt C., Wilander E., Hayden D., O'brien T., Johnson K. (1987). Amyloid fibrils in human insulinoma and islets of Langerhans of the diabetic cat are derived from neuropeptide-like protein also present in normal islets. Proc. Natl. Acad. Sci. USA.

[8] Wimalawansa S.J. (1997). Amylin, caleitonin, caleitonin gene-related peptide, and adrenomdullin: a peptide superfamily. Crit. Rev. Neurobiol..

[9] Schmitz O., Brock B., Rungby J. (2004). Amylin Agonists: A Novel Approach in the Treatment of Diabetes. Diabetes.

[10] Kapurniotu A. (2001). Amyloidogenicity and cytotoxicity of islet amyloid polypeptide. Biopolymers.

[11] Kayed R., Bernhagen J., Greenfield N., Sweimeh K., Brunner H., Voelter W., Kapurniotu A. (1999). Conformational Transitions of Islet Amyloid Polypeptide (IAPP) in Amyloid Formation in Vitro. J. Mol. Biol..

[12] Padrick S.B., Miranker A.D. (2002). Islet Amyloid: Phase Partitioning and Secondary Nucleation Are Central to the Mechanism of Fibrillogenesis. Biochemistry.

[13] Hull R.L., Westermark G.T., Westermark P., Kahn S.E. (2004). Islet amyloid: a critical entity in the pathogenesis of type 2 diabetes. J. Clin. Endocrinol. Metab..

[14] Lorenzo A., Razzboni B., Weir G.C., Yankner B.A. (1994). Pancreatic islet cell toxicity of amylin associated with type-2 diabetes mellitus. Nature.

[15] Anguiano M., Nowak R., Lansbury P. (2002). Protofibrillar islet amyloid polypeptide permeabilizes synthetic vesicles by a pore-like mechanism that may be relevant to type II diabetes. Biochemistry.

[16] Porat Y., Kolusheva S., Jelinek R., Gazit E. (2003). The human islet amyloid polypeptide forms transient membrane-active prefibrillar assemblies. Biochemistry.

[17] Abedini A., Raleigh D.P. (2005). The Role of His-18 in Amyloid Formation by Human Islet Amyloid Polypeptide. Biochemistry.

[18] Makin O.S., Serpell L.C. (2004). Structural Characterisation of Islet Amyloid Polypeptide Fibrils. J. Mol. Biol..

[19] Higham C.E., Jaikaran E., Fraser P.E., Gross M., Clark A. (2000). Preparation of synthetic human islet amyloid polypeptide (IAPP) in a stable conformation to enable study of conversion to amyloid-like fibrils. FEBS. Lett..

[20] Jaikaran E., Higham C.E., Serpell L.C., Zurdo J., Gross M., Clark A., Fraser P.E. (2001). Identification of a novel human islet amyloid polypeptide β-sheet domain and factors influencing fibrillogenesis. J. Mol. Biol..

[21] Goldsbury C., Goldie K., Pellaud J., Seelig J., Frey P., Müller S.A., Kistler J., Cooper G., Aebi U. (2000). Amyloid fibril formation from full-length and fragments of amylin. J. Struct. Biol..

[22] Tatarek-Nossol M., Yan L., Schmauder A., Tenidis K., Westermark G., Kapurniotu A. (2005). Inhibition of hIAPP amyloid-fibril formation and apoptotic cell death by a designed hIAPP amyloid-core-containing hexapeptide. Chem. Biol..

[23] Scrocchi L.A., Chen, Waschuk S., Wang F., Cheung S., Darabie A.A., McLaurin J., Fraser P.E. (2002). Design of peptide-based inhibitors of human islet amyloid polypeptide fibrillogenesis. J. Mol. Biol..

[24] Yan L., Tatarek-Nossol M., Velkova A., Kazantzis A., Kapurniotu A. (2006). Design of a mimic of nonamyloidogenic and bioactive human islet amyloid polypeptide (IAPP) as nanomolar affinity inhibitor of IAPP cytotoxic fibrillogenesis. Proc. Natl. Acad. Sci. USA.

[25] Kapurniotu A., Schmauder A., Tenidis K. (2002). Structure-based design and study of non-amyloidogenic, double N-methylated IAPP amyloid core sequences as inhibitors of IAPP amyloid formation and cytotoxicity. J. Mol. Biol..

[26] Guo L.w., Qu N. (2006). Chemical-induced unfolding of cofactor-free protein monitored by electrochemistry. Anal. Chem..

[27] Vestergaard M., Kerman K., Saito M., Nagatani N., Takamura Y., Tamiya E. (2005). A rapid label-free electrochemical detection and kinetic study of Alzheimer's amyloid beta aggregation. J. Am. Chem. Soc..

[28] Vestergaard M., Kerman K., Tamiya E. (2007). An overview of label-free electrochemical protein sensors. Sensors.

[29] Zhang W., Fan C., Sun Y., Li G. (2003). An electrochemical investigation of ligand-binding abilities of film-entrapped myoglobin. Biochim. Biophys. Acta. Gen. Sub..

[30] Huang Y., Liu L., Shi C., Huang J., Li G. (2006). Electrochemical analysis of the effect of Ca^2+^ on cardiolipin–cytochrome c interaction. Biochim. Biophys. Acta. Gen. Sub..

[31] Xiao H., Wang J., Chen G., Li G. (2007). Electrochemical evaluation of self-disassociation of PKA upon activation by cAMP. Langmuir.

[32] Xiao H., Zhou H., Chen G., Liu S., Li G. (2007). Interaction between inducible nitric oxide synthase and calmodulin in Ca^2+^-free and -bound forms. J. Proteome Res..

[33] Anguiano M., Nowak R., Lansbury P.T.J. (2002). Protofibrillar islet amyloid polypeptide permeabilizes synthetic vesicles by a pore-like mechanism that may be relevant to type II diabetes. Biochemistry.

[34] Green J.D., Goldsbury C., Kistler J., Cooper G.J., Aebi U. (2004). Human amylin oligomer growth and fibril elongation define two distinct phases in amyloid formation. J. Biol. Chem..

